# Herbal Products as Complementary or Alternative Medicine for the Management of Hyperglycemia and Dyslipidemia in Patients with Type 2 Diabetes: Current Evidence Based on Findings of Interventional Studies

**DOI:** 10.1155/2024/8300428

**Published:** 2024-07-10

**Authors:** Hossein Farhadnejad, Niloufar Saber, Asal Neshatbini Tehrani, Mitra Kazemi Jahromi, Ebrahim Mokhtari, Mostafa Norouzzadeh, Farshad Teymoori, Golaleh Asghari, Parvin Mirmiran, Fereidoun Azizi

**Affiliations:** ^1^ Nutrition and Endocrine Research Center Research Institute for Endocrine Sciences Shahid Beheshti University of Medical Sciences, Tehran, Iran; ^2^ Student Research Committee Ahvaz Jundishapur University of Medical Sciences, Ahvaz, Iran; ^3^ Department of Nutrition School of Allied Medical Sciences Ahvaz Jundishapur University of Medical Sciences, Ahvaz, Iran; ^4^ Endocrinology and Metabolism Research Center Hormozgan University of Medical Sciences, Bandar Abbas, Iran; ^5^ Department of Nutrition School of Public Health Iran University of Medical Sciences, Tehran, Iran; ^6^ Department of Community Nutrition Faculty of Nutrition Sciences and Food Technology National Nutrition and Food Technology Research Institute Shahid Beheshti University of Medical Sciences, Tehran, Iran; ^7^ Endocrine Research Center Research Institute for Endocrine Sciences Shahid Beheshti University of Medical Sciences, Tehran, Iran

## Abstract

Type 2 diabetes (T2D) is known as a major public health problem with a noticeable adverse impact on quality of life and health expenditures worldwide. Despite using routine multiple pharmacological and nonpharmacological interventions, including diet therapy and increasing physical activity, controlling this chronic disease remains a challenging issue, and therapeutic goals are often not achieved. Therefore, recently, other therapeutic procedures, such as using herbal products and functional foods as complementary or alternative medicine (CAM), have received great attention as a new approach to managing T2D complications, according to the literature. We reviewed the existing evidence that supports using various fundamental medicinal herbs, including cinnamon, saffron, ginger, jujube, turmeric, and barberry, as CAM adjunctive therapeutic strategies for T2D patients. The current review addressed different aspects of the potential impact of the abovementioned herbal products in improving glycemic indices and lipid profiles, including the effect size reported in the studies, their effective dose, possible side effects, herbs-drug interactions, and their potential action mechanisms.

## 1. Introduction

Type 2 diabetes (T2D) is a serious public health issue [1], with the current Global Burden of Disease (GBD) dataset projecting a rise in global T2D prevalence to 7079 individuals per 100,000 by 2030, signifying a persistent upward trend worldwide [1]. In Iran, both men and women have reported T2D prevalence rates exceeding 10% [2], and it is estimated that by 2030, approximately 9.2 million Iranians will be affected by T2D [3]. The hallmark feature of individuals with diabetes is the inability to regulate blood glucose levels effectively, leading to disruptions in the glycemic profile, which serve as the basis for metabolic and cardiovascular complications [4, 5]. Moreover, dyslipidemia is a prevalent complication of diabetes, further linking T2D to an elevated risk of mortality [6]. Complications arising from uncontrolled diabetes, such as dysglycemia and dyslipidemia, can advance to more severe issues, encompassing both micro- and macrovascular complications. These complications significantly diminish the quality of life and may pose life-threatening risks [7, 8].

For treating metabolic disorders such as hyperglycemia and dyslipidemia in diabetic patients, routine interventions involve pharmacological therapies as well as nonpharmacological strategies such as dietary modifications and increased physical activity. However, while modern Western medicine relies on chemical drugs, which may inevitably cause various side effects [9], controlling T2D remains challenging, with therapeutic goals often unmet [10]. Therefore, complementary or alternative medicine (CAM) approaches, such as incorporating herbal products and functional foods, have gained significant interest as a new approach for better managing T2D and its complications [11, 12].

Herbal medicinal products and functional foods, due to their biological characteristics, can serve as effective nutraceuticals and supplementary treatments alongside modern medicine for managing various aspects of T2D, such as glycemic control and lipid profile improvement [12–14]. Various studies have focused on the possible beneficial effects of several key herbs, including cinnamon, saffron, ginger, jujube, turmeric, and barberry, on glycemic indices and lipid profiles in T2D. These studies examined factors such as fasting blood glucose (FBG), hemoglobin A1c (HbA1c), serum insulin, reducing insulin resistance, and improving lipid profiles, triglycerides (TGs), total cholesterol (TC), low-density lipoprotein cholesterol (LDL-C), and high-density lipoprotein cholesterol (HDL-C) [12, 15–22]. Generally, the findings of most of these studies agree on their antihyperglycemic and antilipidemic properties; however, there are some differences and inconsistencies in the results of the studies.

Since hyperglycemia and dyslipidemia are the two primary metabolic challenges in T2D management [23], routinely monitored through biomarkers such as HbA1c, FBG, insulin, TGs, TC, and LDL-C, there is high importance in investigating herbal products as adjunctive therapies alongside conventional treatment. Therefore, this review aims to provide a comprehensive overview of current evidence regarding the potential glycemic control and antilipidemic benefits of the abovementioned herbs, as well as their proposed mechanisms of action in T2D.

## 2. Main Text

### 2.1. Research

Although various herbal products may be consumed by T2D patients without knowing their effectiveness or possible negative effects, in the present study, the criterion for choosing herbal products to investigate their importance in T2D management was the existence of several studies (at least 2-3 clinical trials in addition to experimental studies) to help us provide valid and valuable content based on these findings. We reviewed and summarized the relevant studies, including *in vitro* and experimental studies, clinical trials with proper design, and high-quality review articles and meta-analyses published from 1990 to 2024. Various databases, including Web of Science (ISI), MEDLINE/ PubMed, and Scopus were used to search for relevant published papers on this topic. Based on the results of our comprehensive search, we identified herbal products that were extensively investigated in previous studies to control glycemic indices and improve lipid profile levels in patients with T2D. Considering the existence of assessable evidence, the herbal products selected for the present study included cinnamon, ginger, saffron, jujube, turmeric, and barberry.

We first briefly introduced the selected herbal products and their properties and then reviewed the evidence on their potential efficacies in improving glycemic indices and lipid profiles in T2D from interventional studies and clinical trials. In the current study, we focused on various aspects of the consumption of herbal products in T2D patients, including their effective dose, possible side effects, drug-herb interactions, and the percentage of changes observed in the metabolic indices mentioned above in response to the consumption of herbal products. In addition, we explored the potential mechanistic pathways through which these herbal products may exert beneficial effects on lipid and glycemic control in T2D.

### 2.2. Herbal Products and Their Effect on Glycemic Indices and Lipid Profile in T2D

#### 2.2.1. Cinnamon

Cinnamon is a reputed spice and a member of the Lauraceae family, which has been consumed for many years as a flavoring agent in many foods and beverages worldwide [24]. It is considered a treatment for diabetes in Ayurvedic and Chinese medicine [25]. So far, approximately 250 species of cinnamon are recognized. The four main cinnamon species include *True or Ceylon cinnamon (Cinnamomum (C) zeylanicum), C. loureiroi, C. burmanni, and C. aromaticum* [26]. The beneficial effects of cinnamon have been proven for various diseases, including metabolic syndrome, T2D, hyperlipidemia, and arthritis [27]. Moreover, cinnamon is widely acknowledged for its antidiabetic, antioxidant, anti-inflammatory, and antibacterial properties [28].

Several studies indicated the improving effect of cinnamon on glycemic indicators in patients with T2D [29]. Based on Mang et al., after four months of supplementation with a purified aqueous cinnamon extract, a 10.3% reduction in fasting plasma glucose was observed [30]. A study by Vafa et al. [12] reported a moderate improvement in glycemic indicators after eight weeks of supplementation with cinnamon in patients suffering from T2D. Also, based on a meta-analysis of 10 RCTs, cinnamon doses of 120 mg/d–6 g/d for 4–18 weeks could decrease fasting plasma glucose up to −24.59 mg/dL [31]. Furthermore, an updated systematic review and dose-response meta-analysis of clinical trials reported that consuming cinnamon significantly reduced the levels of FBS (standard mean difference (SMD: −1.32; 95% CI: −1.77, −0.87)), homeostatic model assessment for insulin resistance (SMD: −1.32; 95% CI: −1.77, −0.87), and HbA1c (SMD: −0.67; 95% CI: −1.18, −0.15) in patients with T2D. However, cinnamon did not have a significant effect on insulin levels (SMD: −0.17; 95% CI: −0.34, 0.01).

Based on the findings from several randomized clinical trials (RCTs), cinnamon could also improve dyslipidemia in subjects with T2D. Khan et al. [32] revealed that consuming different doses of cinnamon could decrease serum TGs, LDL-C, and TC in people with T2D. Anderson et al. [33] reported a lowering effect of cinnamon on TC and LDL-C after two months of supplementation in subjects with elevated serum glucose. A systematic review and meta-analysis study also indicated the lowering effect of cinnamon on TC, LDL-C, and TG levels and the increasing impact on the level of HDL-C [31]; it can lower TC (−15.60 mg/dL), LDL-C (−9.42 mg/dL), and TG (−29.59 mg/dL) while increasing HDL-C (1.66 mg/dL) [31].

The most effective doses and duration of cinnamon supplementation, contributing to glycemic and lipid control, are ≤1.5 gr/day and >8 weeks [34]. According to research, no side effects have been observed when using cinnamon as a flavoring agent in the daily diet at doses of less than 6 g per day. However, some side effects, mostly self-limited allergic reactions, have been reported with large doses and prolonged periods of consumption [35, 36]. Several herb-drug interactions are considered for cinnamon intake, including antidiabetics, antibiotics, anticoagulants, antifungals, antineoplastic agents, antiretroviral agents, anxiolytics, estrogens, hepatotoxic agents, immunosuppressants, and sympathomimetics [37].

Undoubtedly, one of the important characteristics of cinnamon is its insulin-mimetic activity [38, 39]. Cinnamon could increase the activity of insulin receptors by inducing the insulin-receptor kinases and inhibiting the insulin-receptor phosphatases [39]. The inhibition of *α*-amylase and *α*-glucosidase enzymes is another main strategy by which cinnamon exerts its antidiabetic activity. When prepared under high pressure and using the decoction method, *Cinnamomum zeylanicum* released bioactive compounds including benzoic acid, (E)-cinnamaldehyde, trans-cinnamic acid, eugenol, and o-methoxycinnamaldehyde, which are responsible for the inhibition of *α*-glucosidase and management of hyperglycemia [40]. Another imperious bioactive compound of cinnamon is cinnamaldehyde, which can decrease plasma glucose levels. Increasing the expression of proteins involved in glucose transport, insulin signaling pathway, and dyslipidemia is another mechanism by which bioactive compounds of cinnamon administer their antidiabetic effects. Cinnamon extracts could induce the enzymes involved in glycogen synthase, increase glucose uptake primarily by stimulating glucose transporter 4 (GLUT4), and inhibit glycogen synthase kinase-3*β* [41–43]. Furthermore, oxidative stress and consequent tissue damage are the main reasons for insulin resistance and T2D. *C. zeylanicum* has a high antioxidant and free radical scavenging activity [44]. Thus, using that as an antioxidant can increase the level of antioxidant enzymes such as superoxide dismutase (SOD), catalase (CAT), and glutathione (GSH). Consequently, this leads to a reduction of the lipoperoxidation (LPO) level and the index of apoptotic, as well as the removal of reactive oxygen species (ROS) [45]. Moreover, several studies have shown the anti-inflammatory effects of cinnamon [46, 47]. Hydroxycinnamaldehyde as a cinnamon component could inhibit the activity of nuclear factor kappa B (NF-*κ*B) and prohibit the production of nitric oxide [48]. An inflammatory signaling cascade including Src/spleen-tyrosine kinase (Src/Syk) affected by the ethanolic extract of the cinnamon resulted in a decrease in its activity [49]. Inhibition of the TNF-*α* gene is another mechanism by which cinnamon exerts its anti-inflammatory mechanism through the modulation of JNK, p38, ERK1/2 activation, and I*κ*B*α* inhibition [50].

The positive effects of cinnamon on dyslipidemia could also be explained by several mechanisms. It is suggested that cinnamon can activate the different isoforms of peroxisome proliferator-activated receptors (PPARs), which are mainly expressed in brown adipose tissue and liver. Activation of these receptors resulted in declining plasma TGs and increasing levels of HDL-C [51]. Furthermore, the phenolic ingredient found in cinnamon bark extract, known as cinnamate, could ameliorate hyperlipidemia. It seems that this bioactive component has a direct effect on lipid metabolism through the hindrance of the activity of hepatic *β*-hydroxy *β*-methylglutaryl-CoA (HMG-CoA) reductase, a key enzyme in the cholesterol synthesis pathway, thus lowering the level of cholesterol and impeding lipid peroxidation via increasing the activity of the hepatic antioxidant enzymes [52, 53]. Cinnamon could also decline the level of TGs mainly due to its lipolytic activity [54]. Cinnamon could also have a significant inhibitory effect on the oxidation of copper-mediated LDL-C and restrain macrophages from LDL-C phagocytosis. Suppressing the activity of cholesteryl ester transfer protein (CETP) is another cinnamon potential mechanism to improve dyslipidemia [55].

#### 2.2.2. Saffron

*Crocus sativus* L. is a plant with red stigmas in dried form, and it is used as a spice called saffron or crocus [56]. There are several active compounds derived from the plant extract, including crocins, picrocrocin, crocetin, and safranal, with crocins making up around 10% of the total content [57]. Crocins have also demonstrated antidiabetic and lipid-lowering effects [58].

Saffron has positive effects on the glycemic status and lipid profile of people with T2D, according to findings from RCTs and review articles. Barari et al. [59] revealed that saffron extract, along with aerobic training in men with T2D, can increase Apo-A1 regardless of training. This finding implies the positive effect of saffron on diabetic subjects with dyslipoproteinemia. Based on another RCT, saffron consumption in overweight/obese patients with T2D led to a decrease in FPG, TGs, and insulin levels after eight weeks of supplementation [60]. A recent systematic review and meta-analysis reported that supplementation with saffron, administered within a range of 5 mg/day to 1 g/day, resulted in notable reductions in both FPG levels (with a weighted mean difference (WMD) of −8.42 mg/dL) and levels of HbA1c (with a WMD of −0.22%) when compared to a placebo. However, no significant differences were noted for insulin levels, QUICKI, and HOMA-IR [61]. Another systematic review and meta-analysis of 15 RCTs showed that saffron extract was associated with reductions in HbA1c (WMD: −0.35%), FBG (WMD: −26.90), and TC (WMD: −9.29). Similarly, crocin supplementation was found to decrease HbA1c (WMD: −0.43%) and FBG levels (WMD: −14.10) [62].

The effective dose of saffron reported by clinical trials is 30–50 mg/day [63]. Saffron, along with its volatile and nonvolatile constituents, including crocin, safranal, and crocetin, showed some embryonic malformations at high doses in animal models, but no side effects were observed at pharmacological doses [64]. Saffron has been associated with potential herb-drug interactions, particularly with medications such as selective serotonin reuptake inhibitors (SSRIs), monoamine oxidase inhibitors (MAOIs), fertility agents, Alzheimer's medications, antihypertensives, and anticoagulants [65].

Saffron exhibits hypoglycemic effects by promoting glucose uptake, inhibiting intestinal glucose absorption [66], increasing insulin sensitivity via AMPK activation [67], and inducing translocation of GLUT4 [68]. The flavonoids and terpenes in saffron also inhibit *α*-glucosidase to reduce glucose absorption [69]. Several studies indicate that saffron has the potential to alleviate diabetes complications. The compound crocin can improve diabetic neuropathy by reducing oxidative stress, stimulating PI3K/Akt pathways, and treating diabetic retinopathy in microglial cells [70].

The lipid-lowering effects of saffron are attributed to several mechanisms, including activating pancreatic lipase [71], antioxidant activity, increasing adiponectin, inducing PPAR-*α*, and modifying heat shock proteins [72]. The compound crocin increases HDL-C by inhibiting CETP [73]. Crocetin lowers cholesterol indirectly by inhibiting the oxidation of TG [74] and LDL-C [75]. Saffron possesses anti-inflammatory effects by inhibiting cyclooxygenase enzymes and prostaglandin E2 [76], hindering the production of TNF-*α*, blocking NF-*κ*B, and increasing the activity of histone deacetylase activity [77–79]. Saffron's antioxidant properties are related to the radical scavenging abilities of crocin, crocetin, and safranal, which can donate hydrogen atoms to free radicals. Saffron extracts can also decrease malondialdehyde levels, a marker of lipid peroxidation caused by reactive oxygen species [80–82].

#### 2.2.3. Ginger

Ginger, scientifically known as *Zingiber officinale*, is a traditional herb from the Zingiberaceae family that has been used for centuries to treat a variety of health conditions, including T2D [83]. Ginger has also been found to possess potent antioxidant and anti-inflammatory properties, which can protect against oxidative stress and inflammation that contribute to the development of various diseases [84]. The active compounds in ginger responsible for its health benefits include shogaols, gingerols, paradols, zingerone, and zingiberene [85].

Numerous studies have shown that these compounds have antidiabetic properties, such as antihyperglycemic and lipid-lowering effects, which can be beneficial for individuals with T2D [86]. In addition, the consumption of ginger has been shown to significantly reduce insulin resistance and improve insulin levels. In a systematic review and meta-analysis, ginger consumption was found to improve fasting insulin and HOMA-IR, which are important markers of insulin sensitivity [19]. Several studies have demonstrated that ginger may have the potential to improve dyslipidemia in individuals with T2D. For instance, according to a meta-analysis, ginger supplementation significantly improved lipid profile, resulting in notable reductions in TC, LDL-C, and TG levels, along with a significant increase in HDL-C in people with T2D [19]. A recent meta-analysis of 10 RCTs showed a significant reduction in FBS and HbA1c following ginger supplementation in patients with T2D [87]. Specifically, the meta-analysis found that ginger supplementation significantly lowered FBS levels by an average of 18.81 mg/dL and decreased HbA1c levels by an average of 0.57% [87]. Specifically, this meta-analysis found that ginger supplementation significantly reduced TG by a mean of −24.80 mg/dL, TC by −8.22 mg/dL, and LDL-C by −6.66 mg/dL, while increasing HDL-C by a mean of 1.34 mg/dL [19]. However, another meta-analysis reported no significant influence on lipid profile parameters, including TG, TC, LDL, and HDL levels following ginger supplementation [87]. While the available evidence suggests that ginger may have the potential to improve lipid profiles in individuals with T2D, the results have been somewhat mixed. Further research is still needed to establish the optimal dose and long-term effects.

Ginger supplementation, typically administered in the form of powdered ginger capsules, has shown effectiveness at doses ranging between 1.2 and 4 grams per day. Clinical trials evaluating ginger have lasted up to a maximum of 12 weeks, consistently reporting it as well-tolerated and generally safe [88]. In some cases, only mild side effects, such as slight abdominal discomfort, have been reported [89]. However, ginger may not be suitable for all populations, as it can interact with blood-thinning medications and potentially exacerbate conditions such as gastroesophageal reflux [89, 90]. Interestingly, animal studies suggest that ginger may have synergistic benefits when used in combination with certain antidiabetic medications or other natural products, such as cinnamon [91], potentially enhancing their glucose-lowering effects [92].

Several mechanisms have been proposed to explain the antihyperglycemic effect of ginger. Ginger increases GLUT4 expression and glucose uptake in adipocytes and muscle cells [93], inhibits *α*-amylase and *α*-glucosidase enzymes, thereby reducing glucose absorption [94], and enhances insulin secretion by modulating KATP channels and GLP-1 pathway [93, 95]. Ginger's bioactive compounds, such as 6-gingerol promote glucose by increasing glucose-stimulated insulin secretion via cAMP, PKA, and CREB activation in pancreatic islets [93], while also regulating Rab27A GTPase for improved insulin exocytosis [93]. These mechanisms collectively improve insulin sensitivity, glucose uptake, and glucose regulation in peripheral tissues, which is beneficial for individuals with hyperglycemia [96]. The beneficial effects of ginger on dyslipidemia may be attributed to its ability to enhance lipid metabolism by modulating the expression of specific marker enzymes, such as retinoid-binding protein (RBP) [97], regulating carbohydrate conversion to TG by modulating the expression of carbohydrate response element-binding protein (ChREBP) [98], inhibiting cholesterol biosynthesis by targeting HMG-CoA reductase, and promoting cholesterol conversion to bile acids by activating cholesterol 7*α*-hydroxylase [99, 100]. Niacin, a nutrient found in ginger, may also play a role in reducing serum TG levels, increasing the clearance of very low-density lipoprotein-cholesterol (VLDL-C), enhancing hepatic uptake of LDL-C, and inhibiting cholesterol synthesis [101].

#### 2.2.4. Ziziphus Jujube

Ziziphus jujube fruit (*Ziziphus jujuba* Mill.), commonly known as red date, belongs to the Rhamnaceae family. The favorable effects of *Ziziphus jujube* on the pathogenesis of several chronic diseases, such as cancer [102], liver disease [103], and neural function [104] account for its health-promoting properties. The jujube fruit has been identified as a natural sedative containing numerous bioactive agents, including alkaloids, amino acids, polyphenols, polysaccharides, fatty acids, saponins, triterpenic acids, and nucleotides, such as cAMP and cGMP, which contribute to its therapeutic properties. However, these functions can be influenced by the type of cultivation, processing, and storage of *Z. jujuba*, which should be considered [105]. Interestingly, studies conducted on diabetic rat models have demonstrated that jujube supplementation can lead to favorable effects on lipid profiles and oxidative stress markers [106].

The available research exploring *Ziziphus jujube's* effects on T2D patients comprises three RCTs conducted in Iran [107–109]. According to Farhadnejad et al., consumption of 30 g of jujube per day during 12 weeks of intervention period has resulted in a decrease of approximately 11.36% in FPG, a 13.59% decline in TG, a 7.46% reduction in TC, and a 7.65% lowering in LDL-C levels [107]. In another trial, T2D patients in the intervention group who consumed 30 g of dried *Z. vulgaris* daily for 12 weeks experienced significant reductions in insulin levels, HOMA-IR, ApoB100 levels, and hs-CRP. In addition, the *Z. vulgaris* group showed increased values for QUICKI and ApoA-I levels versus the controls at the end of the intervention period [108]. Yazdanpanah et al. showed that the infusion of 10 gr in 100 mL boiling water of jujube thrice daily for 12 weeks along with a restricted-calorie diet led to a decrease in HbA1c, TC, TGs, LDL-C, LDL-C to HDL-C ratio, and TC to HDL-C ratio [109].

The results of interventional studies show that the percentage of changes in the serum levels of glycemic indices and lipid profile in response to *Ziziphus jujube* consumption has been variable. A review of accumulated evidence showed that *Ziziphus jujube* supplementation reduced FBG (range: −0.02 to −12.00%), HbA1c (range: −0.80 to −3.54%), and insulin (−18.81%) levels. *Ziziphus jujube* also has a significant effect on insulin resistance, as evidenced by a decrease in HOMA-IR by 17.46%. It improves the lipid profile by reducing TC (range: −22 to 3.95%), TG (range: −7.62 to −14.00%), and LDL-C (range: −0.13 to −7.56%), while elevating HDL-C levels (range: 0.80 to 3.00%). Studies typically administered 30 g of *Ziziphus jujube* per day for 12 weeks as an effective dose [107–109]. Farhadnejad et al. [107] and Irannejad et al. [108] reported no side effects associated with *Ziziphus jujube* supplementation, while in the study by Yazdanpanah et al. [109], some participants were excluded due to experiencing hypotension. Furthermore, none of the studies reported any interactions between *Ziziphus jujube* supplementation and commonly used drugs or supplements for T2D.

While the precise mechanisms underlying Jujube's health benefits are not fully understood, several potential explanations for its effects have been proposed. Evidence suggests that certain compounds found in jujube, including saponin glycosides, could play a role in improving glycemic profile by increasing insulin secretion and thereby reducing insulin resistance [110]. Moreover, the polyphenol content of jujube can reduce carbohydrate absorption by inhibiting intestinal alpha-glycosidase and hepatic glucose synthesis [109]. Chlorogenic acid, for instance, binds to the intestinal glucose transporters, thus reducing carbohydrate absorption [111]. Furthermore, stabilizing the structure of lipoprotein lipase through enhancements in apoprotein-A can lead to improved glucose hemostasis due to enhanced mitochondrial function [108].

Jujube's bioactive components, such as chlorogenic acid and polysaccharides, may also improve insulin resistance and dyslipidemia [112]. Jujube's phytosterols and water-soluble fiber, such as inulin, have the potential to decrease fat absorption in the gut and enhance lipid metabolism [113]. Moreover, short-chain fatty acids produced during the fermentation of jujube in the intestine can restrict hepatic cholesterol metabolism [114]. *Furthermo*re, jujube's ability to reduce inflammation positions it as a potential tool for managing metabolic dysregulation, such as dyslipidemia [115].

#### 2.2.5. Turmeric (*Curcuma longa*)

Turmeric, also known as *Curcuma longa* and a member of the Zingiberaceae family, has been used in both cuisine and traditional medicine for centuries due to its various therapeutic properties [116]. Curcumin, the primary active component of *Curcuma longa* L., has been shown to have anti-inflammatory and antioxidant effects, in addition to its potential therapeutic benefits for various diseases, including diabetes [117].

Numerous studies have demonstrated the antidiabetic properties of curcumin, with several investigations reporting positive effects on glycemic control and lipid profiles in individuals diagnosed with T2D. According to a recent systematic review of 11 RCTs, curcumin was found to have a positive impact on glycemic control in patients with T2D, with significant reductions in FBS, HbA1c, and HOMA-IR [118]. Two recent meta-analyses also observed that curcumin therapy significantly reduced FBS and HbA1c and may help improve insulin resistance and glycemic control in T2D patients. Furthermore, these meta-analyses noted that curcumin therapy resulted in a decrease in serum TG and TC levels [119, 120].

Based on the results reported in meta-analyses, the effect of turmeric and its effective ingredient (curcumin) consumption on improving glycemic indices and lipid profile in T2D has been noticeable. Tian et al. found that curcumin supplementation resulted in significant reductions in FBG (−8.85 mg/dL), HbA1c (−0.54%), TG (mean change of −18.97 mg/dL), and TC (−8.91 mg/dL) with curcumin supplementation [119]. Another meta-analysis of 59 RCTs reported that turmeric supplementation reduced FBG levels by an average of 4.60 mg/dL, lowered fasting insulin levels by 0.87 *μ*IU/mL, and decreased HbA1c by 0.32%. It also improved insulin resistance, with an average reduction of 0.33 in HOMA-IR scores across the trials [121]. A separate meta-analysis comprising 64 RCTs found that curcumin supplementation led to lower levels of TC by a mean of 3.99 mg/dL, TG by 6.69 mg/dL, and LDL-C by 4.89 mg/dL. However, HDL-C levels were higher by a mean of 1.80 mg/dL with curcumin supplementation compared to placebo [121]. While the effect sizes varied across meta-analyses, likely due to differences in the included studies (e.g., turmeric dose), the overall evidence suggests that turmeric supplementation can significantly improve glycemic control and lipid profile in T2D patients.

The effective doses of curcumin used in these studies ranged from 300 mg/day to 2,100 mg/day of turmeric powder containing curcuminoids, with intervention durations typically spanning from 8 to 12 weeks. Turmeric supplementation, in the form of standardized powder and extract containing curcuminoids, has demonstrated safety for human consumption. Even doses as high as 8 g/day can be tolerated without significant adverse effects [122]. However, it is important to note that some adverse effects have been reported, including upset stomach, nausea, and diarrhea [122]. Turmeric has been shown to interact with some medications by affecting cytochromes P450, leading to potential alterations in the pharmacokinetics of drugs such as anticoagulants, antibiotics, antidiabetics, cardiovascular drugs, anticancer drugs, and antidepressants [123–125].

Curcumin improves glycemic control in T2D through multiple mechanisms. It increases PPAR activity to lower FBS, promotes glycolysis and insulin release while suppressing hepatic gluconeogenesis, stimulates insulin secretion and glucose uptake by upregulating glucose transporters (GLUT4, GLUT2, and GLUT3), decreases hepatic glucose production via AMPK activation and inhibition of glucose 6-phosphate kinase [126–128], increases GLP-1 and inhibits DPP-4 [129], and enhances insulin sensitivity by upregulating adiponectin [130]. Moreover, curcumin's antioxidant and anti-inflammatory properties, mediated by inhibiting NF-*κ*B and activating PPAR-*γ* pathways, contribute to improving *β*-cell function and glucose metabolism in T2D [126, 131].

The lipid-lowering effects of turmeric and curcumin are likely due to their ability to regulate multiple pathways involved in lipid, cholesterol, and fatty acid metabolism. Proposed mechanisms include increasing lipoprotein lipase activity for lipid hydrolysis [126], enhancing cholesterol catabolism via cholesterol 7*α*-hydroxylase while inhibiting cholesterol synthesis by HMG-CoA reductase, modulating LDL receptors and dietary cholesterol absorption, inhibiting fatty acid synthase, and promoting fatty acid *β*-oxidation [132]. Curcumin specifically reduces TG by modulating pathways regulated by PPARs [133], CETP [134], and lipoprotein lipase [126].

#### 2.2.6. Barberry (*Berberis vulgaris* L.)

Barberry, also known as Berberis, is a plant that belongs to the Berberidaceae family and has a long history of use in traditional medicine [135]. Barberry fruit contains several biologically active compounds, such as berberine, berbamine, oxyacanthine, protoberberine, and polyphenols [136]. Due to the presence of these compounds, the potential positive effects of barberry on several chronic diseases, such as metabolic syndrome [137] and T2D [138], have been demonstrated. Also, the beneficial effects of barberry in the form of juice [139] or extract [22], including anti-inflammatory, hypoglycemic, and lipid-lowering effects have been reported in previous investigations.

A meta-analysis of 14 RCTs with 1,068 participants found that berberine is effective in treating hyperglycemia and dyslipidemia in diabetic patients without causing serious adverse effects [140]. Shidfar et al. found that in diabetic patients, daily consumption of 3 g of barberry for 12 weeks led to significant reductions in FBS, insulin levels, HOMA-IR, TC, LDL-C, and TG levels while having no significant effect on HDL-C levels [22]. The efficacy of berberine was evaluated in patients with T2D by Yin et al., who found significant reductions in FBS, HbA1c, and postprandial blood glucose (PBG) levels, suggesting that berberine has an effect similar to that of metformin [141]. In a study conducted by Di Pierro and colleagues, significant improvements in HOMA-IR and insulin levels were observed in patients who took berberine over a 90-day treatment period [142]. Another study conducted on 46 diabetic subjects found that daily consumption of 200 ml of barberry juice for eight weeks led to significant reductions in FBS, TC, and TG levels [139].

Recent meta-analyses have focused on the effectiveness of barberry and its unique compound called berberine in improving various metabolic abnormalities in T2D [143–145]. According to these comprehensive reports, berberine significantly reduces FPG by 0.82–0.86 mmol/L, HbA1c by 0.63–0.73%, and 2hPG by 1.16–1.26 mmol/L in individuals with T2D. Berberine also enhances insulin sensitivity, as evidenced by reductions in fasting insulin levels by a mean difference of around −2.05 and HOMA-IR by a mean difference of −0.71. Regarding lipid parameters, berberine supplementation has been shown to improve lipid profiles, effectively lowering LDL-C by 0.56–0.86 mg/dL, TC by 0.57–0.64 mg/dL, and TG by 0.47–0.5 mg/dL, as well as increasing HDL-C levels by 0.14–0.17 mg/dL [143–145].

Studies have evaluated the efficacy of berberine supplementation in various forms, including extract, fruit, or juice. Low and medium doses of berberine (1-2 g/day) have demonstrated effective results, with better efficacy observed in patients undergoing interventions lasting 8 to 12 weeks. According to a meta-analysis, the efficacy of berberine diminishes with usage beyond 90 days and daily doses exceeding 2 g [146]. Berberine is generally considered safe and typically does not have significant interactions with medications [147]. However, caution is advised with antidiabetic drugs (hypoglycemia risk), antihypertensives (hypotension risk), and anticoagulants/antiplatelets (bleeding risk) [148]. Gastrointestinal side effects, such as diarrhea, bloating, and constipation, were the most commonly reported adverse events. Despite transient digestive side effects, recent findings suggest that berberine is generally well-tolerated and a safe complementary or alternative option for managing T2D [143]. Importantly, its beneficial effects on glycemic control and lipid profiles occurred without a notable rise in adverse events or hypoglycemia risk, whether berberine was used alone or with other oral hypoglycemic medications [144].

Barberry may exert its hypoglycemic effect through various mechanisms, including increasing insulin sensitivity, modulating gut microbiota, activating the adenosine AMPK pathway, promoting intestinal GLP-1 secretion, stimulating glycolysis in peripheral tissue cells, inhibiting gluconeogenesis in the liver, increasing glucose transporter expression [149], and decreasing the activity of *α*-glucosidase, which may lead to the inhibition of carbohydrate absorption [150]. Moreover, berberine can inhibit mitochondrial function by reducing oxygen consumption and increasing the AMP/ATP ratio, resulting in upregulation of glucose utilization. The antidiabetic effects of barberry may also be partially attributed to the antioxidant properties of its anthocyanin compounds. These anthocyanins can protect pancreatic beta cells against oxidative stress by modulating inflammatory pathways. They also stimulate insulin secretion from the pancreas and activate the AMPK enzyme, which enhances glucose uptake in muscle cells and contributes to reducing circulating glucose concentrations in the body [151].

Berberine and anthocyanin polyphenols from barberry have been proposed to beneficially modulate lipid levels through several mechanisms. Berberine improves hepatic function and bile secretion, inhibits cholesterol absorption by blocking HMG-CoA reductase [22, 152], and activates PPAR-*α* to reduce TG [153]. Anthocyanins downregulate lipogenic gene expression and upregulate energy expenditure genes in adipose/muscle tissue to lower LDL-C and TG [154]. Berberine also decreases PCSK9 expression [155], upregulates LDL-C receptor expression [156], and increases cholesterol excretion/bile acid production [157]. It also limits fatty acid synthesis via AMPK stimulation [158].

This study comprehensively focused on the potential efficacy of herbal products for ameliorating metabolic abnormalities in T2D. Specifically, we identified crucial herbs, their effective dosages, impacts on glycemic and lipid parameters, potential adverse effects, drug-herb interactions, and underlying mechanisms of action on metabolic indicators in T2D. The findings present a comprehensive resource on using herbal products as CAM alongside standard therapies for the management of T2D, providing researchers and healthcare professionals access to existing evidence on herbal remedies as an adjunct treatment approach. However, the current study has some limitations. The evidence regarding the effectiveness of some herbal products, such as *Ziziphus jujube*, in improving metabolic conditions in T2D is not yet conclusive. Therefore, further large-scale clinical trials are needed to show or confirm the effectiveness of *Ziziphus jujube* consumption in controlling T2D complications. In addition, most interventional studies examining the potential effects of using certain herbal products in T2D have been conducted in countries of the Middle East, North Africa, East Asia, and South America, such as China, Iran, and Brazil. As a result, the generalizability of the results from these studies to other communities, such as those in Western countries, may be limited.

## 3. Conclusions

The current review suggested that according to the results of experimental research and clinical trials, certain herbal products such as cinnamon, saffron, ginger, turmeric, and barberry can be considered potentially effective agents for T2D management and the prevention of its complications via beneficial impacting glycemic indices and lipid profiles. However, further research is recommended to confirm the possible significant effects of certain herbs, including jujube, as supplementary treatments for T2D. In addition, there are knowledge gaps in some aspects of herbal medicine. Herbal products contain a variety of compounds and ingredients, making it challenging to accurately identify all beneficial, nonactive, and potentially toxic components. Therefore, more studies are needed to address these knowledge deficiencies and clarify the therapeutic potential of herbal medicine in T2D management.

## Data Availability

No data were used to support the study.

## Abbreviations

AMPK: AMP-activated protein kinase
ACC: Acetyl-CoA carboxylase
CAM: Complementary and alternative medicine
CAT: Catalase
CETP: Cholesteryl ester transfer protein
ChREBP: Carbohydrate response element-binding protein
COX-2: Cyclooxygenase-2
DPP-4: Dipeptidyl peptidase-4
ERK1/2: Extracellular signal-regulated protein kinases 1 and 2
FBG: Fasting blood glucose
GBD: Global Burden of Disease
GLP-1: Glucagon-like peptide 1
GSH: Glutathione
GLUT4: Glucose transporter type 4
HbA1c: Hemoglobin A1c
HMG-CoA: *β*-Hydroxy *β*-methylglutaryl-CoA
HOMA-IR: Homeostatic model assessment of insulin resistance
hs-CRP: C-reactive protein
I*κ*B*α*: Inhibitor of nuclear factor kappa-B
IL-6: Interleukin 6
ISI: Web of Science
JNK: c-Jun N-terminal kinase
LPO: Lipoperoxidation
MAPKs: Mitogen-activated protein kinases
MDA: Malondialdehyde
NF-*κ*B: Nuclear factor kappa B
NOS: Nitric oxide synthase
LDL-C: Low-density lipoprotein cholesterol
HDL-C: High-density lipoprotein cholesterol
PCSK9: Proprotein convertase subtilisin/kexin type 9
PPAR: Peroxisome proliferator-activated receptor
QUICKI: Quantitative insulin sensitivity check index
ROS: Reactive oxygen species
SOD: Superoxide dismutase
Src/Syk-: Src/spleen-tyrosine kinase-
RBP: Retinoid-binding protein
RCTs: Randomized clinical trials
TAC: Total antioxidant capacity
T2D: Type 2 diabetes
TC: Total cholesterol
TGs: Triglycerides
TNF: Tumor necrosis factor
VLDL: Very low-density lipoprotein.
